# Mental disorders around cancer diagnosis and increased hospital admission rate - a nationwide cohort study of Swedish cancer patients

**DOI:** 10.1186/s12885-018-4270-4

**Published:** 2018-03-27

**Authors:** Jianwei Zhu, Arvid Sjölander, Katja Fall, Unnur Valdimarsdottir, Fang Fang

**Affiliations:** 1 Department of Medical Epidemiology and Biostatistics, Karolinska Institutet, Solnavägen 1, Stockholm, Sweden; 2 Clinical Epidemiology and Biostatistics, School of Medical Sciences, Örebro University, SE-701 82 Örebro, Sweden; 3 Department of Epidemiology, Harvard T.H. Chan School of Public Health, 677 Huntington Avenue, Boston, MA USA; 4 Faculty of Medicine, Center of Public Health Sciences, School of Health Sciences, University of Iceland, Sæmundargata 2, Reykjavík, Iceland

**Keywords:** Mental disorder, Cancer, Hospital admission, Comorbidity, Survival analysis

## Abstract

**Background:**

Whether the emotional distress around cancer diagnosis is associated with the long-term outcomes and care utilization is unknown. We aimed to examine the association of mental disorders around cancer diagnosis with the hospital admission rates of cancer patients thereafter.

**Methods:**

We conducted a nationwide cohort study including 218,508 cancer patients diagnosed in Sweden during 2004–2009 and followed them from 90 days after cancer through 2010. We used a clinical diagnosis of stress-related mental disorders from 90 days before to 90 days after cancer diagnosis as the exposure. We studied first all hospital admissions and then separately three common admissions, including external injuries, infections, and cardiovascular diseases. The Cox model was used to estimate hazard ratios (HRs) with 95% confidence intervals (CIs).

**Results:**

Four thousand one hundred five patients received a diagnosis of stress-related mental disorders around the cancer diagnosis, and experienced a 35% increased rate of any hospital admission during follow-up (HR: 1.35, 95%CI: 1.28–1.41) as well as hospital admissions for external injuries (HR: 1.89, 95%CI: 1.67–2.14), infections (HR: 1.28, 95%CI: 1.08–1.52), and cardiovascular diseases (HR: 1.16, 95%CI: 1.03–1.30). Similar association was noted for most common cancer types.

**Conclusions:**

These data suggest that cancer patients diagnosed with a stress-related mental disorder immediately before or after cancer diagnosis are subsequently at increased risk of hospital admissions for major comorbidities of cancer.

**Electronic supplementary material:**

The online version of this article (10.1186/s12885-018-4270-4) contains supplementary material, which is available to authorized users.

## Background

The diagnostic work-up for a suspected cancer finally leading to a definite cancer diagnosis is a highly stressful period for patients. Indeed, a considerable increase in a range of psychiatric disorders, including depression, anxiety, and other stress-related reactions has been observed among cancer patients during the months before [1–3] and after [1, 4] cancer diagnosis. In addition to the swift deterioration in the psychological spheres of the patients’ quality of life, it is possible that this rise in psychiatric disorders around the time of cancer diagnosis leads to further health consequences during the disease course.

Psychiatric disorders in the general population have been associated with elevated risks of injuries [5], cardiovascular diseases [6] and increased susceptibility for infections [7], with corresponding increase in healthcare use and medical costs [8]. Although these associations have not to date been specifically explored among cancer patients, our previous studies indicate that the rise in psychiatric disorders around the cancer diagnosis [1] coincides with a rise in infections and injuries [9] as well as cardiovascular diseases [10]. In our most recent work, we documented an increased risk of cancer-specific mortality among patients with first-onset psychiatric disorders in response to their cancer diagnosis [11]. Similarly, we here aim to explore the potential impact of mental disorders diagnosed immediately before or after a cancer diagnosis on the subsequent rate of hospital admissions for common comorbidities of cancer - namely, external injuries, infections, and cardiovascular diseases.

## Methods

### Study design

We conducted a prospective cohort study based on the Swedish Cancer Register, including all adult patients (30 years and above) with a first primary cancer diagnosed between January 1st 2004 and December 31st 2009 (*N* = 251,214). Information available in this register includes date of and age at diagnosis, sex, cancer type, cancer stage, and the Swedish national identity number. Cancer types were coded according to the 7th Swedish revision of the International Classifications of Diseases (ICD) codes [12]. Since 2004, the register has also collected relatively complete information on cancer stage at the time of diagnosis, except for hematological cancers and central nervous system (CNS) tumors. Patients with CNS tumors (*N* = 6061) were not included in the present study because the occurrence of mental disorders among these patients might be physiologically related to the lesion of the CNS [13, 14]. Patients that had emigrated before cancer diagnosis (*N* = 6255) were excluded from the study cohort. The individually unique national identify numbers, assigned to all Swedish residents, allowed record linkages between the study cohort and other nationwide population and health registers [15].

### Ascertainment of mental disorders

Mental disorders were ascertained by cross-linking all cancer patients to the Swedish Patient Register. In Sweden, all hospital discharge records have been collected in the Patient Register since 1987 and > 80% of outpatient visits for hospital-based specialist care since 2001 [16]. In this register, all diagnoses are coded from 1997 according to the 10th Swedish revision of the ICD codes. We focused on the mental disorders that are commonly diagnosed among cancer patients [17], for which highly increased risks have been reported immediately before and after cancer diagnosis [1]. We included depression (ICD10: F30-F39), anxiety (ICD10: F40-F41), stress reaction or adjustment disorder (ICD10: F43), mental and behavioral disorders due to psychoactive substance use (ICD10: F10-F16, F18-F19), and somatoform/conversion disorder (ICD10: F44-F45). Because these disorders have been suggested as potentially related to severe psychological stress [18–21], we used “stress-related mental disorders” as a collective term for all the selected disorders.

In Sweden, the median waiting time, between the first referral to a specialist and the start of cancer treatment, is around 2 months [22]. The symptoms of cancer, as well as the suspicion of a potential cancer disease, probably start even earlier than the first referral for most patients. The psychological distress in relation to these might be reflected by the increasing risk of stress-related mental disorders during the 10 months before cancer diagnosis, especially during the months immediately before and after cancer diagnosis [1]. We therefore aimed to use stress-related mental disorders as indices of severe stress response toward the cancer diagnostic process and the eventual diagnosis. We used a hospital visit, whether inpatient or outpatient, with a stress-related mental disorder as either the primary or a secondary diagnosis from 90 days before to 90 days after cancer diagnosis as the primary exposure of interest. We used all other mental disorders, including organic (ICD10: F00–09), tobacco abuse (ICD10: F17), schizophrenia (ICD10: F20–29), affective (ICD10: F30–31/F34–39), neurotic (ICD10: F42/48), physiological/physical factors related (ICD10: F50–59), personality (ICD10: F60–69), retardation (ICD10: F70–79), psychological development (ICD10: F80–89), and unspecified (ICD10: F90–99) disorders from 90 days before to 90 days after cancer diagnosis as the secondary exposure.

We categorized cancer patients with no hospital visit for any mental disorders during this time window as the reference group.

### Ascertainment of hospitalizations after cancer diagnosis

We followed all cancer patients from 90 days after diagnosis until death or December 31st 2010, whichever occurred first. Patients who died within the first 90 days after their cancer diagnosis were excluded, leaving 218,508 patients (91.47%) in the final analyses. Hospital admissions of these patients during follow-up were also identified from the Patient Register. Information on dates of admission and discharge, as well as the main discharge diagnosis of each hospitalization was retrieved. Consecutive hospital admissions were treated as one admission event. We first performed analysis for any hospital admission as a proxy for overall healthcare utilization. We then performed separate analyses for three types of common hospital admissions, including external injuries (ICD10: S00-S99, T00-T36, T51-T79, T89-T95, T97-T98.2, T98.4-T99) - both unintentional injuries (ICD10: V01-X59, Y85-Y86) and self-harm (ICD-10: X60-X84,Y870) [9], infections (ICD10: A00-A99, B00-B99), and cardiovascular diseases (CVD, ICD10: I00-I99), as well as for hospital admissions of different durations (< 4 days, 4–10 days, and > 10 days).

### Statistical analysis

We used Cox regression to assess the association of mental disorders diagnosed from 90 days before to 90 days after a cancer diagnosis with the subsequent rate of hospital admissions. Because patients might be repeatedly admitted, a clustered sandwich estimator was used to account for intra-individual correlation. We performed the analysis first for all cancer patients (excluding patients with hematological cancers because we adjusted for cancer stage in this analysis and cancer stage is not applicable for these patients) and then separately for patients with the most common cancer types, including breast, prostate, colorectal, lung, melanoma, kidney or bladder, and hematological cancers. Esophageal, liver, and pancreatic cancers were combined into one group of severe cancers.

In all statistical models, we used age at follow-up as the underlying timescale and adjusted for age at cancer diagnosis (as a continuous variable), sex, calendar year of cancer diagnosis (as a continuous variable), cancer type (see Additional file 1: Table S1), cancer stage at diagnosis (except for hematological cancers), educational level (≥9 years or < 9 years), and history of mental disorders more than 90 days before cancer diagnosis (yes or no). In the analysis of hematological cancers, we further adjusted for cancer subtype, including Hodgkin lymphoma, non-Hodgkin lymphoma, myeloma, and leukemia. Information on cancer stage was ascertained by the TNM or FIGO records from the Cancer Register, including localized (T-localized/N0/M0 or FIGO 0, I), local spread (T-advanced/N0/M0 or FIGO II), regional spread (any T/N+/M0 or FIGO III), advanced (any T/any N/M+ or FIGO IV), and unknown cancers. History of any mental disorders more than 90 days before cancer diagnosis was ascertained from the Patient Register. Patients with at least one hospital visit (inpatient or outpatient) concerning a mental disorder from year 2001 to 90 days before cancer diagnosis were classified as having a history of mental disorder.

To assess potential effect modifiers of the studied associations, we further stratified the analyses by age group (≤65, 66–75, and > 75 years), sex, calendar period of diagnosis (2004–2006, 2007–2009), cancer stage, educational level, and history of mental disorders. We assessed the statistical significance of these effect modifications by introducing interaction terms between the exposure and these variables to the Cox regression.

Mental disorders around the time of cancer diagnosis could be associated with the type of cancer treatment, leading to different hospital admission rates later on. We therefore performed a sensitivity analysis for cancers commonly treated by surgeries, including prostate, lung, and colorectal cancers. We repeated the main analyses for these cancers, after additionally adjusting for surgical treatment (yes or no). To explore the role of individual stress-related mental disorders, we performed separate analysis on the risk of hospital admissions in relation to different stress-related mental disorders around cancer diagnosis. Finally, to assess whether the results were sensitive to the choice of exposure time window and the inclusion of patients with previous mental disorders, we performed a sensitivity analysis including only cancer patients without any mental disorders before cancer diagnosis and using only mental disorders diagnosed during the 90 days after cancer diagnosis as the exposure.

All the statistical analyses were carried out in SAS9.4 (SAS Institute, North Carolina, United States) and Stata14.4 (StataCorp LP).

## Results

From 90 days before to 90 days after cancer diagnosis, 4105 patients (1.88%) had a hospital visit concerning a stress-related mental disorder and 3594 (1.64%) had a hospital visit concerning other mental disorders (Table 1).

**Table 1:** Baseline characteristics of 218,508 cancer patients that were diagnosed during 2004–2009 in Sweden, *N* (%)

	Unexposed patients	Stress-related mental disorders^a^	Other mental disorders^b^
Sex			
Male	113,099 (53.65)	1845 (44.95)	1688 (46.97)
Female	97,710 (46.35)	2260 (55.05)	1906 (53.03)
Age at diagnosis, years			
≤ 65	89,975 (42.68)	2303 (56.10)	1162 (32.33)
66–75	60,538 (28.72)	996 (24.26)	822 (22.87)
> 75	60,296 (28.60)	806 (19.63)	1610 (44.80)
Calendar period at diagnosis			
2004–2006	104,917 (49.77)	1870 (45.55)	1531 (42.60)
2007–2009	105,892 (50.23)	2235 (54.45)	2063 (57.40)
Educational level			
> 9 years	126,879 (60.19)	2547 (62.05)	1787 (49.72)
≤ 9 years	83,686 (39.70)	1550 (37.76)	1804 (50.19)
Missing	244 (0.12)	8 (0.19)	3 (0.083)
Previous mental disorders			
No	201,345 (95.51)	2147 (52.30)	1876 (52.20)
Yes	9464 (4.49)	1958 (47.70)	1718 (47.80)
Cancer stage^c^			
Localized	98,192 (46.58)	1550 (37.76)	1273 (35.42)
Local spread	26,512 (12.58)	535 (13.03)	540 (15.03)
Regional spread	23,368 (11.08)	636 (15.49)	568 (15.80)
Advanced	15,981 (7.58)	369 (8.99)	324 (9.02)
Unknown	33,022 (15.66)	710 (17.30)	664 (18.48)
Not applicable	13,734 (6.51)	305 (7.43)	225 (6.26)

^a^Stress-related mental disorders included depression, anxiety, stress reaction and adjustment disorder, mental and behavioral disorders due to psychoactive substance use, and somatoform/conversion disorder

^b^Other mental disorders included organic, tobacco abuse, schizophrenia, affective, neurotic, physiological/physical factors related, personality, retardation, psychological development, and unspecified disorders

^c^Patients with missing or unclear information of TNM/FIGO were classified as ‘Unknown’. Hematological cancers were classified as ‘Not applicable’

Cancer patients with a stress-related mental disorder around cancer diagnosis had greater number of hospital admissions during follow-up (median: 2; range: 0–68), compared to patients with other mental disorders (median: 1; range: 0–75) or without any mental disorders (median: 1; range: 0–57) (*p* < 0.01, Table 2) around cancer diagnosis. Similar differences were observed for most of the common cancers, including prostate, breast, colorectal, renal and bladder, and hematological cancers.

**Table 2:** Number of hospital admissions during follow-up

	All patients	Unexposed patients		Stress-related mental disorders^a^		Other mental disorders^b^		
	*N*	*N*	Median (Range)	*N*	Median (Range)	*N*	Median (Range)	*P* ^d^
Any cancer	218,508	210,809	1 (0–57)	4105	2 (0–68)	3594	1 (0–75)	< 0.01
Prostate cancer	50,393	49,458	1 (0–45)	521	2 (0–33)	414	1 (0–20)	< 0.01
Breast cancer	33,734	32,466	1 (0–45)	706	1 (0–37)	562	1 (0–18)	< 0.01
Lung cancer	12,676	11,932	2 (0–57)	338	2 (0–31)	406	2 (0–18)	0.31
Colorectal cancer	25,733	24,650	1 (0–43)	518	2 (0–68)	565	1 (0–75)	< 0.01
Melanoma	10,905	10,695	0 (0–27)	125	1 (0–14)	85	1 (0–10)	< 0.01
Hematological cancers	14,264	13,734	2 (0–43)	305	2 (0–28)	225	1 (0–30)	< 0.01
Renal/Bladder cancer	14,666	14,093	2 (0–36)	277	2 (0–28)	296	2 (0–22)	< 0.05
Severe cancers^c^	4223	3989	2 (0–38)	135	1 (0–19)	99	1 (0–17)	< 0.01

^a^ Stress-related mental disorders included depression, anxiety, stress reaction and adjustment disorder, mental and behavioral disorders due to psychoactive substance use, and somatoform/conversion disorder

^b^ Other mental disorders included organic, tobacco abuse, schizophrenia, affective, neurotic, physiological/physical factors related, personality, retardation, psychological development, and unspecified disorders

^c^ Severe cancers included esophageal, liver, and pancreatic cancers

^d^ Nonparametric tests were used to test the median and range differences

Compared to the unexposed group, patients with a stress-related mental disorder around cancer diagnosis had a 35% increased rate of any hospital admission during follow-up; whereas patients with a hospital visit for other mental disorders had a 7% increased rate (Table 3). Both rate increases were more pronounced for hospital admissions of > 10 days’ duration compared to admissions of shorter durations. For stress-related mental disorders, the increased rate was highest for external injury-related hospitalizations (unintentional injuries: hazard ratio [HR] = 1.85, 95% confidence interval [CI]: 1.62–2.12; intentional injuries: HR = 6.64, 95%CI: 1.09–40.31), followed by infection-related, and cardiovascular disease-related hospitalizations. For other mental disorders, an increased rate was noted for external injury-related and infection-related hospitalizations.

**Table 3:** Associations of mental disorders around cancer diagnosis with hospital admission, among all cancer patients

	Stress-related mental disorders; HR (95% CI)^a^	Other mental disorders^b^; HR (95% CI)
Any hospital admission	1.35 (1.28–1.41)	1.07 (1.01–1.13)
Duration of admission		
< 4 days	1.25 (1.18–1.33)	1.01 (0.93–1.10)
4–10 days	1.32 (1.25–1.40)	1.04 (0.98–1.10)
> 10 days	1.63 (1.53–1.73)	1.24 (1.15–1.33)
Main discharge diagnosis		
External injury	1.89 (1.67–2.14)	1.36 (1.20–1.55)
Infection	1.28 (1.08–1.52)	1.19 (1.00–1.42)
Cardiovascular disease	1.16 (1.03–1.30)	0.91 (0.80–1.03)

^a^Stress-related mental disorders included depression, anxiety, stress reaction and adjustment disorder, mental and behavioral disorders due to psychoactive substance use, and somatoform/conversion disorder; *HR* Hazard ratio, *CI* Confidence interval; models adjusted for age at cancer diagnosis, sex, calendar period of cancer diagnosis, cancer type, cancer stage at diagnosis, educational level, and history of mental disorders more than 90 days before cancer diagnosis; patients without any mental disorders from 90 days before to 90 days after cancer diagnosis were used as the reference group

^b^Other mental disorders included organic, tobacco abuse, schizophrenia, affective, neurotic, physiological/physical factors related, personality, retardation, psychological development, and unspecified disorders

A stress-related mental disorder around cancer diagnosis was associated with an increased rate of hospital admission among almost all cancer types although the associations were not statistically significant for melanoma and severe cancers (Table 4). Statistically significantly increased rate of hospital admission for external injuries was noted for all cancer types except melanoma, lung and severe cancers. Statistically significantly increased rate of hospital admission for infections was observed for breast, colorectal, and hematological cancers, whereas statistically significantly increased rate of hospital admissions for cardiovascular diseases was only noted for colorectal cancer.

**Table 4:** Associations of stress-related mental disorders around cancer diagnosis with hospital admission, among different cancer types^a^

Cancer types	Main discharge diagnosis of hospitalization			
	Any hospital admission; HR (95% CI)^b^	External injury; HR (95% CI)	Infection; HR (95% CI)	Cardiovascular disease; HR (95% CI)
Prostate cancer	1.56 (1.39–1.76)	2.33 (1.71–3.18)	0.91 (0.58–1.45)	1.15 (0.87–1.52)
Breast cancer	1.44 (1.28–1.61)	1.54 (1.18–2.01)	1.51 (1.05–2.17)	1.29 (0.99–1.69)
Lung cancer	1.30 (1.13–1.49)	1.43 (0.84–2.41)	0.72 (0.37–1.40)	0.99 (0.66–1.48)
Colorectal cancer	1.46 (1.27–1.68)	1.89 (1.44–2.49)	1.60 (1.08–2.39)	1.39 (1.06–1.83)
Melanoma	1.05 (0.79–1.40)	0.96 (0.42–2.21)	NA	1.41 (0.83–2.40)
Hemetological cancers	1.42 (1.22–1.65)	1.99 (1.26–3.16)	1.47 (1.05–2.07)	0.97 (0.66–1.44)
Renal/Bladder cancer	1.37 (1.16–1.61)	2.01 (1.31–3.07)	1.54 (0.98–2.42)	1.15 (0.78–1.71)
Severe cancers^c^	1.13 (0.93–1.37)	1.12 (0.38–3.36)	1.87 (0.81–4.35)	1.17 (0.57–2.41)

^a^Stress-related mental disorders included depression, anxiety, stress reaction and adjustment disorder, mental and behavioral disorders due to psychoactive substance use, and somatoform/conversion disorder

^b^*HR* Hazard ratio, *CI* Confidence interval; models adjusted for age at cancer diagnosis, sex, calendar period of cancer diagnosis, cancer type, cancer stage at diagnosis, educational level, and history of mental disorders more than 90 days before cancer diagnosis; patients without any mental disorders from 90 days before to 90 days after cancer diagnosis were used as the reference group

^c^Severe cancers included esophageal, liver, and pancreatic cancers

The magnitude of the estimated associations were similar for all patients, regardless of age, sex, calendar period, educational level, history of mental disorders, or cancer stage (Table 5). However, we did observe a statistically significant difference between sexes, with stronger associations for the males, for any hospital admission, hospital admission for external injuries, and hospital admission for cardiovascular diseases (all *P*-values for interaction: < 0.05). We also observed statistically significant differences across age groups, with stronger associations for the young patients, for any hospital admission and for hospital admission for external injuries (both *P*-values for interaction: < 0.05). Finally, we observed statistically significant differences across cancer stages, with stronger associations for early stages, for any hospital admission (*P*-value for interaction: < 0.01), although similar patterns were not clear for hospital admissions for specific reasons.

**Table 5:** Associations of stress-related mental disorders around cancer diagnosis with hospital admission, a stratification analysis^a^

	Main discharge diagnosis of hospital admission			
	Any hospital admission; HR (95% CI)^b^	External injury; HR (95% CI)	Infection; HR (95% CI)	Cardiovascular disease; HR (95% CI)
Sex				
Male	1.42 (1.32–1.52)	2.12 (1.76–2.55)	1.35 (1.05–1.72)	1.25 (1.06–1.47)
Female	1.28 (1.20–1.37)	1.68 (1.43–1.97)	1.24 (0.98–1.57)	1.05 (0.89–1.23)
Age at follow-up, years				
≤ 65	1.36 (1.27–1.45)	1.99 (1.56–2.53)	1.44 (1.11–1.87)	1.20 (0.96–1.51)
66–75	1.36 (1.25–1.49)	1.85 (1.47–2.34)	0.98 (0.72–1.35)	1.19 (0.94–1.52)
> 75	1.21 (1.11–1.31)	1.62 (1.36–1.94)	1.25 (0.92–1.70)	1.01 (0.86–1.19)
Calendar period at diagnosis				
2004–2006	1.34 (1.25–1.44)	1.84 (1.57–2.17)	1.15 (0.91–1.45)	1.11 (0.96–1.30)
2007–2009	1.36 (1.28–1.45)	1.93 (1.61–2.31)	1.42 (1.11–1.83)	1.25 (1.03–1.52)
Educational level				
> 9 years	1.34 (1.26–1.43)	1.96 (1.66–2.32)	1.29 (1.04–1.59)	1.09 (0.93–1.29)
≤ 9 years	1.36 (1.27–1.46)	1.75 (1.47–2.08)	1.28 (0.96–1.69)	1.20 (1.01–1.43)
Previous mental disorders				
No	1.32 (1.24–1.41)	1.86 (1.55–2.22)	1.40 (1.11–1.76)	1.01 (0.86–1.19)
Yes	1.34 (1.25–1.43)	1.82 (1.53–2.15)	1.12 (0.87–1.43)	1.28 (1.06–1.53)
Cancer stage^c^				
Localized	1.48 (1.37–1.60)	2.01 (1.67–2.42)	1.15 (0.86–1.55)	1.21 (1.01–1.45)
Local spread	1.27 (1.12–1.43)	1.92 (1.46–2.52)	1.44 (0.95–2.17)	1.36 (1.02–1.82)
Regional spread	1.23 (1.12–1.37)	1.48 (1.06–2.06)	1.03 (0.67–1.61)	1.35 (1.01–1.79)
Advanced	1.08 (0.95–1.22)	1.52 (0.94–2.45)	1.52 (0.80–2.87)	0.78 (0.51–1.21)
Unknown	1.42 (1.27–1.60)	2.07 (1.56–2.73)	0.99 (0.65–1.50)	0.97 (0.73–1.29)

^a^Stress-related mental disorders included depression, anxiety, stress reaction and adjustment disorder, mental and behavioral disorders due to psychoactive substance use, and somatoform/conversion disorder

^b^*HR* Hazard ratio, *CI* Confidence interval; models adjusted for age at cancer diagnosis, sex, calendar period of cancer diagnosis, cancer type, cancer stage at diagnosis, educational level, and history of mental disorders more than 90 days before cancer diagnosis; patients without any mental disorders from 90 days before to 90 days after cancer diagnosis were used as the reference group

^c^Patients with missing or unclear information of TNM/FIGO were classified as ‘Unknown’

In the additional analysis of prostate, lung, and colorectal cancers, we found similar risk elevations of hospital admission by stress-related mental disorders after adjustment for surgical treatment (Additional file 1: Table S2). We further found increased rates of hospital admissions for all individual stress-related mental disorders studied (Additional file 1: Table S3). In the sensitivity analysis for patients without a history of mental disorders before cancer diagnosis, we found very similar associations between mental disorders during the 90 days after cancer diagnosis and increased rate of hospital admission thereafter, as in the main analysis (Additional file 1: Table S4).

## Discussion

In this nationwide cohort study we found that a diagnosis of stress-related mental disorders immediately before and after cancer diagnosis was associated with increased healthcare utilization and morbidity of the cancer patients, indexed by an increased rate of hospital admission. The increased rate was mostly attributable to hospital admissions for external injuries, infections, and cardiovascular diseases. The increased rate was also more pronounced for admissions of longer duration and did not differ by age, sex, calendar period, history of mental disorders, or cancer stage. Although other mental disorders around cancer diagnosis were also associated with a slightly higher rate of hospital admission, the magnitude of rate increase was considerably smaller.

To the best of our knowledge, our study represents the first large-scale investigation to evaluate the influence of a severe stress response during the diagnostic process of cancer on the rate of any hospital admission - as a proxy for overall healthcare use - including admissions for injuries, infections, and cardiovascular diseases. A statistically significantly increased rate for hospital admission was noted for all major cancer types, except for melanoma and cancers with poor prognosis. The reason for the null findings among patients with melanoma and severe cancers is not entirely clear, although lack of statistical power in detecting real associations due to the small numbers of patients included in the analyses and the relatively short follow-up might be a partial explanation. The modestly increased rate of hospital admission due to other mental disorders around cancer diagnosis, in comparison to stress-related mental disorders, might suggest that a severe stress response to cancer diagnosis leads to additionally increased inpatient care use, relative to impaired mental health in general. Taken together, these findings suggest that stress-related mental disorders experienced in close vicinity of cancer diagnosis, as indices of a severe stress response toward the cancer diagnostic process and the diagnosis itself, could lead to increased need for healthcare use and comorbidities, independently of previous mental health status.

Patients with stress-related disorders around the diagnosis of almost all cancer types presented with increased rates of hospital admissions for external injuries, except for melanoma. The stronger associations noted for intentional injuries, compared to unintentional injuries, corroborate and might partly underline earlier findings that cancer patients had increased risks for self-harm including suicide [10]. The clear although slightly weaker associations noted for unintentional injuries are worth noting. Possible underling mechanisms for such associations might include mental distress and worsening of social and physical function in relation to stress-related mental disorders. Finally, both the cognitive impairment and psychiatric symptoms of mental disorders may further contribute to the occurrence of unintentional injuries [23].

An increased rate of infection-related hospital admission was found for stress-related mental disorders around cancer diagnosis. The association was noted for most cancer types, except prostate and lung cancers, and did not differ clearly by age, sex, calendar period, educational level, history of mental disorders, or cancer stage. A similar association was noted for other mental disorders around cancer diagnosis, although with slightly smaller magnitude of rate increase. These findings are indeed biologically plausible. Depression and anxiety have been shown to directly affect the immune system by for example regulating the secretion of pro-inflammatory cytokines [24], and subsequently regulating the response to infections. Psychological stress has specifically been linked with impaired immune response to infectious challenges, leading to increased risk for contagion and prolonged infection episodes [25–27]. Among cancer patients with surgical treatments, psychological stress might also lead to delayed wound healing [28] and increased risk for wound infection [29].

Increased risk for cardiovascular diseases, including coronary artery disease and stroke, have been related to various stress-related mental disorders including depression [30, 31] and anxiety [32, 33]. We observed positive association between stress-related mental disorders around cancer diagnosis and increased rate of cardiovascular disease-related hospital admission, regardless of patient or cancer characteristics. A positive association was also noted for most cancer types, with the exception of lung and hematological cancers, perhaps because of the high baseline rate of cardiovascular diseases among these two cancer groups [34, 35]. Interestingly, the increased rate of cardiovascular disease-related hospital admission appeared to be restricted to stress-related mental disorders, whereas no increased rate was noted for other mental disorders from 90 days before to 90 days after cancer diagnosis, further highlighting the fact that it might be the severe stress response, relative to impaired mental health in general, which led to increased burden of cardiovascular diseases.

The major strength of our study is the large-scale population-based cohort design, the complete follow-up, and the prospectively and independently collected data on mental disorders, cancer, and hospital admissions after cancer diagnosis, minimizing most of the selection and information biases commonly seen in observational studies.

A few limitations should nevertheless be noted. First, we had no information on other potential confounders, including for example lifestyle factors (e.g. smoking and alcohol use) that might be related to both the exposure and the outcome. However, the similar associations noted for different cancer types with or without known links to these lifestyle factors argue against pure explanations by unmeasured confounders. Second, we did not have detailed information on cancer treatment (e.g. radiation or chemotherapies) and disease progression, which could both affect hospital admissions. However, in the sensitivity analysis of prostate, lung, and colorectal cancers, we found similar results after further adjustment for surgical treatment, partially alleviating this concern. Nevertheless, future studies are needed to disentangle the potential role of chemo- and radiation therapies in the associations of stress-related mental disorders and healthcare utilizations. Third, we used the 90 days before cancer diagnosis as a proxy for cancer diagnostic workup, as we had no information on the precise start of the diagnostic process. Although it is difficult to decide whether or not this is the optimal choice of time window, the median waiting time between a first specialist referral and primary cancer treatment is about 2 months in Sweden [22] and there was highly increased risk of stress-related mental disorders during the months immediately before and after cancer diagnosis [1]. Using a clinical diagnosis of stress-related mental disorders captures only the tip-of-the-iceberg of the psychological distress a cancer patient might experience around the diagnosis of cancer [36–38]. Indeed, only 1.88% cancer patients of the present study experienced clinically diagnosed stress-related mental disorders during 90 days before to 90 days after cancer diagnosis, whereas previous studies reported a much higher rate of mental distress [4] and self-reported or clinically evaluated psychiatric disorders [39] among cancer patients. This source of misclassification is however likely to attenuate the real associations between stress-related mental disorders around cancer diagnosis and hospital admission rate. Finally, the present study has an average follow-up of approximately 3 years, whether or not the studied associations would differ during the latter part of cancer survivorship remains to be explored.

## Conclusions

In conclusion, our findings suggest that patients with stress-related mental disorders immediately before or after a cancer diagnosis have an increased rate of hospital admissions after cancer diagnosis. The increased need for hospital admissions among these patients appeared to be mainly due to common comorbidities, including external injuries, infections, and cardiovascular diseases. Our findings thus suggest that better psychological management - through both surveillance and treatment - during the cancer diagnostic workup and immediately after the diagnosis of cancer may help prevent adverse health outcomes and healthcare unitization among this group of patients.

## Additional file

Additional file 1: **Table S1.** Classification of cancer sites and groups. **Table S2.** Associations of mental disorders around cancer diagnosis with the rate of hospital admission, analyses of patients with prostate, lung, or colorectal cancer and after further adjustment for surgical treatment. **Table S3.** Associations of mental disorders around cancer diagnosis with the rate of hospital admission, analyses by specific diagnosis of stress-related mental disorders. **Table S4.** Associations of mental disorders during the 90 days after cancer diagnosis with the rate of hospital admission, analysis among cancer patients without any mental disorders before cancer diagnosis. (DOCX 23 kb)

## Abbreviations

CI: Confidence interval
CNS: Central nervous system
HR: Hazard ratio
ICD: International classifications of diseases
SD: Standard deviation
