# Supplementary material for: Mental disorders around cancer diagnosis and increased hospital admission rate - a nationwide cohort study of Swedish cancer patients
Source: BMC Cancer. 2018 Mar 27;18:322. doi: 10.1186/s12885-018-4270-4 (PMC5870174; doi:10.1186/s12885-018-4270-4)
Supplement: Supplementary file 1 — Table S1. Classification of cancer sites and groups. Table S2. Associations of mental disorders around cancer diagnosis with the rate of hospital admission, analyses of patients with prostate, lung, or colorectal cancer and after further adjustment for surgical treatment. Table S3. Associations of mental disorders around cancer diagnosis with the rate of hospital admission, analyses by specific diagnosis of stress-related mental disorders. Table S4. Associations of mental disorders during the 90 days after cancer diagnosis with the rate of hospital admission, analysis among cancer patients without any mental disorders before cancer diagnosis. (DOCX 23 kb) [file 12885_2018_4270_MOESM1_ESM.docx]

**Mental disorders around cancer diagnosis and increased hospital admission rate**

**- a nationwide cohort study of Swedish cancer patients**

**Jianwei Zhu^1^*****, Arvid Sjölander^1^, Katja Fall^1,2^, Unnur Valdimarsdottir^1,3,4^, Fang Fang^1^**

| **Table S1. Classification of cancer sites and groups.** | |
| --- | --- |
| **Cancer sites and groups** | **Classification of cancer diagnosis (ICD-7*)** |
| Lip, oral cavity and pharynx | 140-148 |
| Digestive cancer | 150-159 |
| Lung and thorax cancer | 160-165 |
| Bone cancer | 196 |
| Skin cancer | 190-191 |
| Soft tissue cancer | 197 |
| Breast cancer | 170 |
| Other female genital cancer | 171-176 |
| Male genital cancer | 177-179 |
| Urinary cancer | 180-181 |
| Eye cancer | 192 |
| Endocrine cancer | 194-195 |
| Hematological malignancies | 200-207 |

* ICD-7 = 7th Swedish revision of the International Classifications of Diseases codes.

| **Table S2. Associations of mental disorders around cancer diagnosis with the rate of hospital admission, analyses of patients with prostate, lung, or colorectal cancer and after further adjustment for surgical treatment** | |  |
| --- | --- | --- |
|  | **Stress-related mental disorders^a^; HR (95% CI)^b^** | |
| **Prostate cancer^c^** | 1.56 (1.39 - 1.75) | |
| **Lung cancer^d^** | 1.32 (1.15 - 1.51) | |
| **Colorectal cancer^e^** | 1.46 (1.27 - 1.69) | |

^a^ Stress-related mental disorders included depression (ICD10: F32-F33), anxiety (ICD: F40-F41), stress reaction and adjustment disorder (ICD10: F43), mental and behavioral disorders due to psychoactive substance use (ICD10: F10-F16, F18-F19), and somatoform/conversion disorder (ICD10: F44-F45).

^b^ HR: Hazard ratio; CI: Confidence interval; models adjusted for age at cancer diagnosis, sex, calendar period of cancer diagnosis, cancer type, cancer stage at diagnosis, educational level, history of mental disorders more than 90 days before cancer diagnosis, and surgical treatment; patients without any diagnosis of mental disorders from 90 days before to 90 days after cancer diagnosis were used as the reference group.

^c^ According to Swedish Classification of care measures (KVA) (<http://www.socialstyrelsen.se/klassificeringochkoder/atgardskoderkva>), prostatectomy was included as the surgical treatment for prostate cancer (KVA: KEC00, KEC01, KEC10, KEC20).

^d^ According to Swedish Classification of care measures (KVA), following surgical treatments for lung cancer were included: lung resection (KVA: GDB10, GDB11, GDB20, GDB21), lobectomy of lung (KVA: GDC00-GDC97), and pulmonectomy (KVA: GDD00-GDD97).

^e^ According to Swedish Classification of care measures (KVA), following surgical treatments for colorectal cancer were included: resection of right-sided semicolon (KVA: JFB30, JFB31), transverse colon (KVA: JFB40, JFB41), left semicolon (KVA: JFB43, JFB44), sigmoid colon (KVA: JFB46, JFB47), other part of colon (KVA: JFB50, JFB51), and colon with rectum (KVA: JFB53, JFB54, JFB60, JFB61, JFB63, JFB64).

| **Table S3. Associations of mental disorders around cancer diagnosis with the rate of hospital admission, analyses by specific diagnosis of stress-related mental disorders^a^** | | | | | |
| --- | --- | --- | --- | --- | --- |
|  | **Stress reaction and adjustment disorder;**  **HR (95% CI)^b^** | **Depression;**  **HR (95% CI)^b^** | **Anxiety;**  **HR (95% CI)^b^** | **Psychoactive substance use;**  **HR (95% CI)^b^** | **Somatoform/conversion disorder;**  **HR (95% CI)^b^** |
| Any hospital admission | 1.35 (1.16 - 1.56) | 1.16 (1.08 - 1.25) | 1.34 (1.21 - 1.49) | 1.72 (1.58 - 1.87) | 1.28 (1.01 - 1.62) |
| Main discharge diagnosis |  |  |  |  |  |
| External injury | 2.68 (1.71 - 4.20) | 1.52 (1.29 - 1.78) | 1.35 (0.96 - 1.92) | 3.63 (2.98 - 4.42) | 0.72 (0.24 - 2.14) |
| Infection | 1.30 (0.78 - 2.14) | 1.06 (0.82 - 1.38) | 1.20 (0.85 - 1.71) | 1.80 (1.31 - 2.47) | 1.34 (0.56 - 3.21) |
| Cardiovascular disease | 1.11 (0.71 - 1.75) | 0.98 (0.84 - 1.15) | 1.40 (1.07 - 1.83) | 1.38 (1.06 - 1.78) | 1.39 (0.69 - 2.79) |

^a^ Stress-related mental disorders included depression, anxiety, stress reaction and adjustment disorder, mental and behavioral disorders due to psychoactive substance use, and somatoform/conversion disorder.

^b^ HR: Hazard ratio; CI: Confidence interval; models adjusted for age at cancer diagnosis, sex, calendar period of cancer diagnosis, cancer type, cancer stage at diagnosis, educational level, and history of mental disorder more than 90 days before cancer diagnosis; patients without any diagnosis of mental disorders from 90 days before to 90 days after cancer diagnosis were used as the reference group.

| **Table S4. Associations of mental disorders during the 90 days after cancer diagnosis with the rate of hospital admission, analysis among cancer patients without any mental disorders before cancer diagnosis** | | |
| --- | --- | --- |
|  | **Stress-related mental disorders; HR (95% CI)^a^** | **Other mental disorders^b^;**  **HR (95% CI)** |
| Any hospital admission | 1.28 (1.17 - 1.40) | 1.11 (1.02 - 1.21) |
| Duration of admission |  |  |
| <4 days | 1.15 (1.02 - 1.30) | 1.03 (0.91 - 1.17) |
| 4-10 days | 1.26 (1.14 - 1.40) | 1.07 (0.97 - 1.19) |
| >10 days | 1.63 (1.46 - 1.83) | 1.34 (1.18 - 1.51) |
| Main discharge diagnosis |  |  |
| External injury | 1.67 (1.30 - 2.14) | 1.61 (1.28 - 2.02) |
| Infection | 1.25 (0.92 - 1.69) | 1.44 (1.09 - 1.92) |
| Cardiovascular disease | 0.96 (0.79 - 1.17) | 0.87 (0.72 - 1.06) |

^a^ Stress-related mental disorders included depression, anxiety, stress reaction and adjustment disorder, mental and behavioral disorders due to psychoactive substance use, and somatoform/conversion disorder; HR: Hazard ratio; CI: Confidence interval; models adjusted for age at cancer diagnosis, sex, calendar period of cancer diagnosis, cancer type, cancer stage at diagnosis, and educational level; patients without any mental disorders within 90 days after cancer diagnosis were used as the reference group.

^b^ Other mental disorders included organic, tobacco abuse, schizophrenia, affective, neurotic, physiological/physical factors related, personality, retardation, psychological development, and unspecified disorders.
